# First case of lupus induced by the Shingrix vaccine: a case report and literature review

**DOI:** 10.1007/s10067-025-07529-2

**Published:** 2025-06-11

**Authors:** Coral Arévalo-Cañas, Juan Arévalo-Serrano, Melchor Álvarez de Mon-Soto

**Affiliations:** Servicio de Medicina Interna, Hospital Universitario Príncipe de Asturias, Alcalá de Henares, Madrid, Spain

**Keywords:** Vaccine, Lupus, DILE

## Abstract

The recombinant zoster vaccine (Shingrix) was recently approved for the prevention of herpes zoster reactivation in adults aged ≥ 50 years. While its effectiveness has been widely demonstrated, its safety profile and potential adverse effects remain uncertain. We report the first case of lupus induced by the Shingrix vaccine. An 85-year-old woman was evaluated in the hospital due to a pleuropericarditis with pleural and pericardial effusion. Test for anti-nuclear antibody was positive at a titer of 1:640 with a homogeneous pattern, as well as for IgG anti-cardiolipin antibodies. Infectious or malignant etiologies were excluded. A diagnosis of Shingrix vaccine-induced lupus was suspected, and a short course of prednisone was initiated. The patient's symptoms resolved within the first two months, and after one year of follow-up, both the antinuclear and anti-cardiolipin IgG antibodies were negative. Although many drug categories have been associated with the development of drug-induced lupus erythematosus (DILE), it remains unclear whether vaccine-induced immune system upregulation could trigger the onset of systemic lupus. However, particularly after the introduction of the SARS-CoV2 vaccine, cases of cutaneous and systemic lupus induced by vaccines have been reported, and the serological pattern appears to be different from that observed in drug-induced lupus. Our case details a critical adverse effect observed in a patient who received the zoster vaccine. This finding is particularly relevant given the ongoing widespread vaccination campaigns and the global public health implications. Vaccine-induced lupus should be suspected following vaccination in the presence of cutaneous or systemic lupus symptoms, particularly serositis or renal involvement, when no other cause can be identified. The diagnosis is supported by positive antinuclear antibodies and other laboratory abnormalities, such as decreased complement levels or positivity for other antibodies, including antiphospholipid antibodies.

**Table:** 

**Key Points** • The varicella-zoster vaccine will be administered to a large percentage of the population, which could lead to an increase in adverse effects that have not yet been described. This article reports the first documented case of lupus induced by the varicella-zoster vaccine. • The diagnosis of Drug-induced lupus erythematosus (DILE) requires a low threshold of suspicion. • Clinical manifestations in DILE are usually milder, and it often presents with general symptoms, arthralgia, serositis, and hematologic abnormalities. • The autoimmune profile of vaccine-induced lupus appears to differ from that of drug-induced lupus, being characterized by a higher frequency of positive antinuclear antibodies (ANA), antiphospholipid antibodies, and hypocomplementemia, with a lower prevalence of anti-histone antibodies.

**Key Points**

• The varicella-zoster vaccine will be administered to a large percentage of the population, which could lead to an increase in adverse effects that have not yet been described. This article reports the first documented case of lupus induced by the varicella-zoster vaccine.

• The diagnosis of Drug-induced lupus erythematosus (DILE) requires a low threshold of suspicion.

• Clinical manifestations in DILE are usually milder, and it often presents with general symptoms, arthralgia, serositis, and hematologic abnormalities.

• The autoimmune profile of vaccine-induced lupus appears to differ from that of drug-induced lupus, being characterized by a higher frequency of positive antinuclear antibodies (ANA), antiphospholipid antibodies, and hypocomplementemia, with a lower prevalence of anti-histone antibodies.

## Introduction

The recombinant zoster vaccine (Shingrix) was recently approved for the prevention of herpes zoster reactivation in adults aged ≥ 50 years. While its effectiveness has been widely demostrated [1, 2], its safety profile and potential adverse effects remain uncertain. The recombinant zoster vaccine contains the varicella zoster virus glycoprotein E (gE) antigen, along with the AS01B adjuvant system, which includes *Quillaja saponaria* Molina fraction 21 (QS-21) and 3-*O*-desacyl-4’-monophosphoryl lipid A (MPL) derived from *Salmonella Minnesota*. AS01B induces a localized and transient activation of the innate immune response, promoting the recruitment and activation of antigen-presenting dendritic cells that carry gE-derived antigens to the draining lymph node. This process leads to the production of gE-specific antibodies and CD4 + T cells. Both QS-21 and MPL work synergistically to enhance the frequency of gE-specific CD4 + T cells [3].

The most common side effects are injection site reactions, and non-severe systemic symptoms have been reported [4]. Trials have not shown an increase in immune-related adverse events compared to placebo, and safety monitoring after licensure are consistent with these results. The most frequently reported potential immune mediated diseases were polymyalgia rheumatica, rheumatoid arthritis, psoriasis, and autoimmune thyroiditis, with incidence rates not differing from those observed in the placebo group [5]. No cases of vaccine-induced lupus have been reported after administration of the recombinant zoster vaccine. We report the first case of lupus induced by the Shingrix vaccine.

## Case report

Informed consent was obtained from the patient for the publication of this case.

**Fig. 1:**
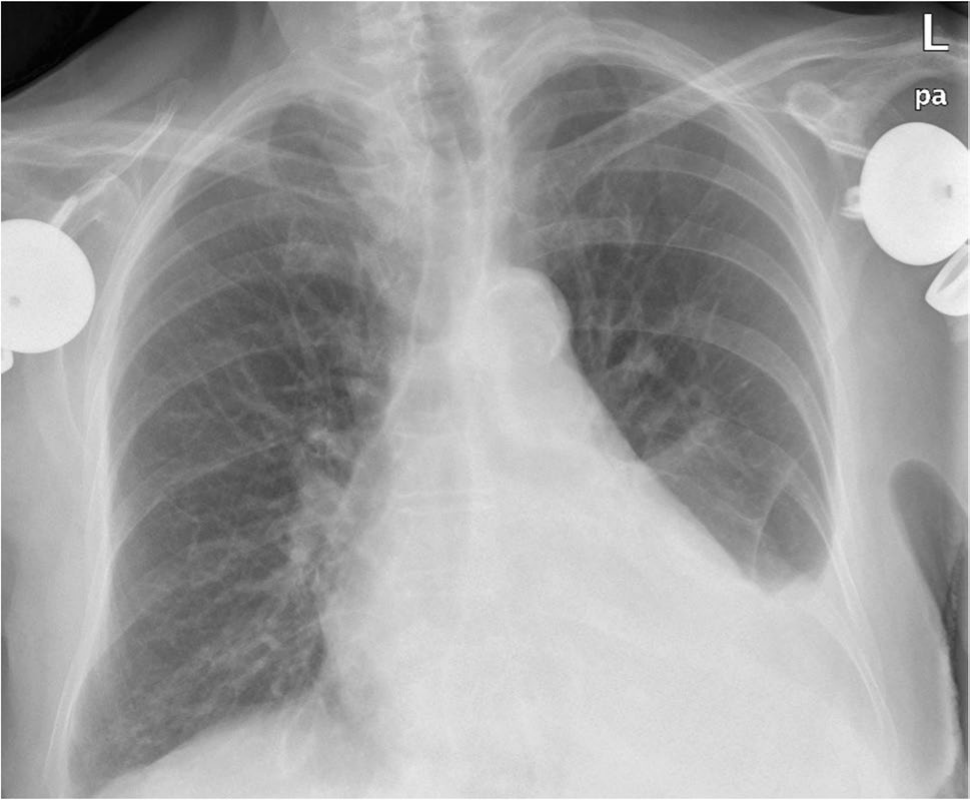
Anteroposterior chest radiograph showing a left-predominant pleural effusion

**Fig. 2:**
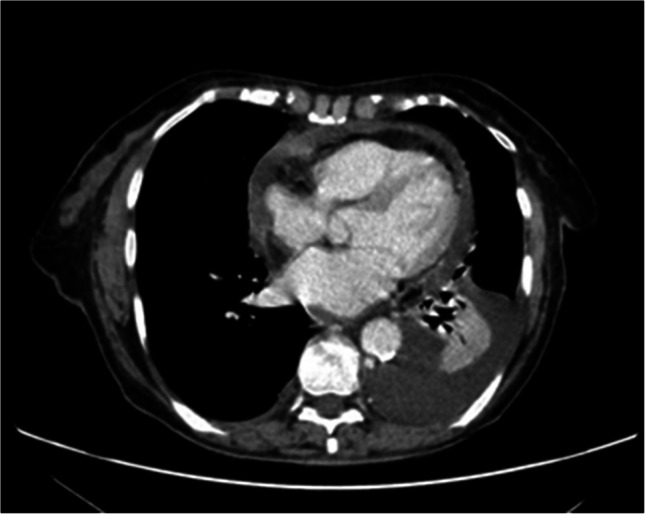
Chest CT showing pleural and pericardial effusion

An 85-year-old woman with medical history of hypertension, hypothyroidism treated with 88 mcg of levothyroxine daily with adequate therapeutic control, and herpes zoster in a single dermatome one year ago, was admitted to our hospital with asthenia, weight loss and a pleuritic chest pain that began two months ago, one week after receiving the first dose of Shingrix vaccination, and her symptoms worsened after the second dose of the vaccine. Upon evaluation, a diagnosis of pleuritis and pericarditis with pleural and pericardial effusion, without hemodynamic compromise (Figs. 1 and 2), was made.

The pleural effusion was consistent with a mononuclear exudate with a normal glucose concentration (320 leukocytes/µl, of which 95% were mononuclear and 5% polymorphonuclear, glucose 101 mg/dl, protein 5,1 g/dl). Infectious or malignant etiologies were excluded (microbiologic tests, pleural fluid cytology and flow cytometry were negative). Thyroid function tests were within normal limits, with a TSH level of 4.07 µIU/mL (reference range: 0.40–5.00 µIU/mL). Both C-reactive protein (CRP) and erythrocyte sedimentation rate (ESR) were elevated in peripheral blood: CRP level was 44 mg/l (normal range < 10 mg/l) and ESR was 40 mm/h (normal range 1–20 mm/h). Initial laboratory results displayed mild thrombocytosis without evidence of cytopenias or other hematological abnormalities. The serological panel revealed a positive anti-nuclear antibody (ANA) at a titer of 1:640 with a homogeneous pattern and positive anti-cardiolipin IgG (46 U.GPL, normal range < 10 U.GPL). The C3 and C4 levels were within normal range, and the rest of serological tests, including anti-histone antibodies and Coombs test, were negative. Positron-emission tomography revealed focal pericardial uptake with no other abnormal findings. Further complementary tests, including computed tomography (CT) of the chest, abdomen, and pelvis, did not reveal any abnormalities.

**Fig. 3:**
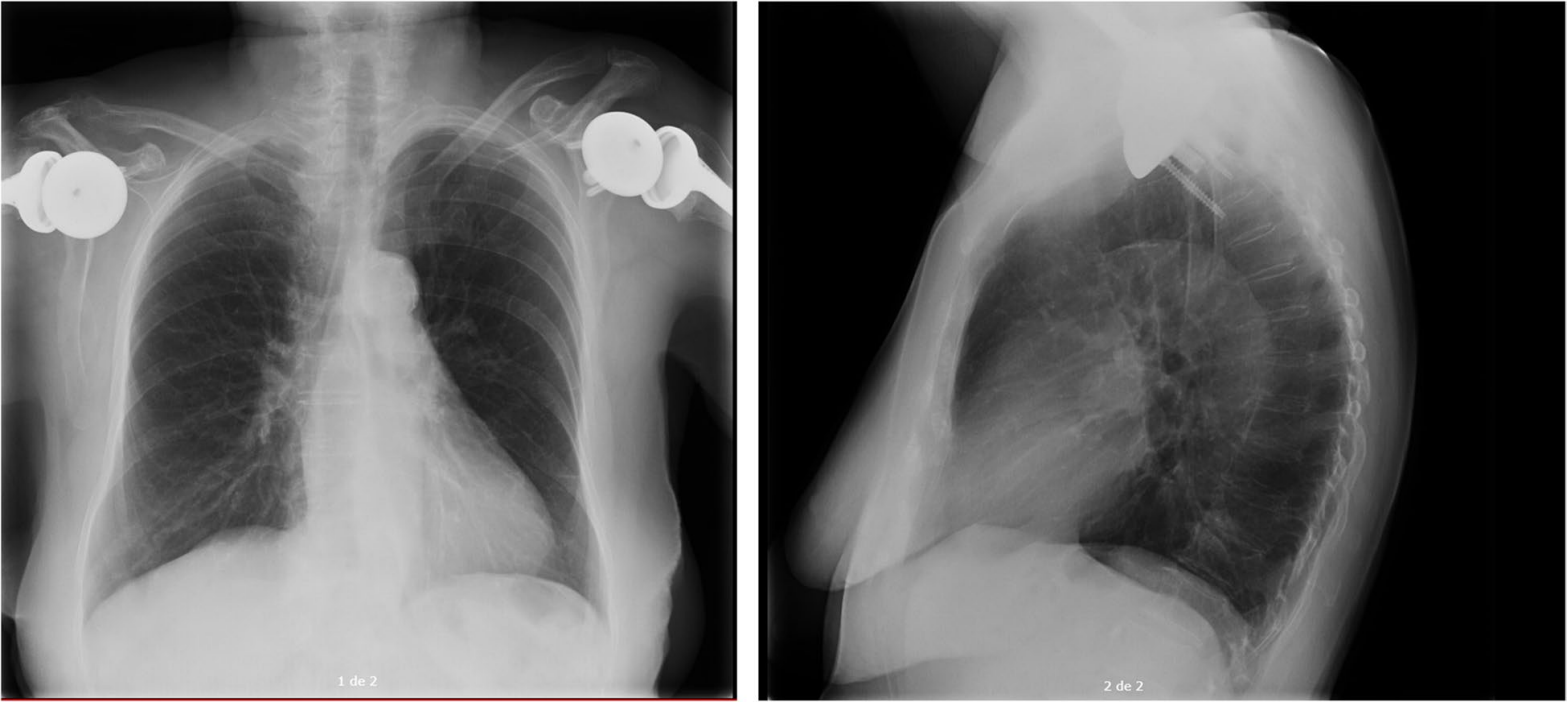
Posteroanterior and lateral chest X-ray after treatment with resolution of pleural and pericardial effusion

A diagnosis of Shingrix vaccine-induced lupus was suspected due to the temporal relationship between vaccine administration and the onset of symptoms, as well as the absence of recent changes in her usual medication regimen. A short course of prednisone (15 mg per day with tapering every five days until discontinuation) was initiated. In follow up the patient's symptoms as well as pleural and pericardial effusion were resolved (Fig. 3), and CRP and ESR levels normalized within the first two months after starting treatment. Likewise, after one year of follow-up, the anti-nuclear and anti-cardiolipin antibodies were negative.

## Review

A comprehensive literature review was conducted to identify published cases of vaccine-induced lupus. The search was performed in PubMed using the keywords'drug-induced lupus'and'vaccine-induced lupus’. Published cases of systemic and cutaneous lupus associated with vaccination published from 01 January 2010 to 24 February 2025 were selected for analysis. Only case reports in English full text were included. There were 16 cases of vaccine-induced lupus reported.

Drug-induced lupus erythematosus (DILE) occurs as a reaction to certain drugs and typically resolves upon discontinuation of the culprit medication. Some drugs may trigger the clinical manifestation of previously asymptomatic lupus, exacerbate a previously diagnosed lupus, or, more commonly, result in the development of drug-induced lupus.

Traditionally, procainamide and hydralazine were the drugs most commonly associated with DILE, although with the introduction of new therapeutic agents, the landscape of this condition has evolved [6]. Today, at least 100 drugs from more than 10 drug categories have been implicated in DILE (Table 1) [6–9]. Although many drug categories have been associated with the development of DILE, whether vaccine-induced immune system upregulation could be a trigger for the onset of systemic lupus remain unclear, and no vaccines have been confirmed to have a role in DILE [10]. However, especially after the COVID-19 pandemic and the introduction of different vaccines against SARS-CoV2, cases of cutaneous [11, 12] and systemic [13–26] lupus induced by vaccine administration have been reported, as well as a case of cutaneous lupus that progressed to systemic lupus [27]. To date, no cases of vaccine-induced lupus have been documented following the administration of the Shingrix vaccine.

**Table 1:** Lupus-inducing drugs

- Antiarrhythmics ⚬ Procainamide ⚬ Quinidine ⚬ Disopyramide ⚬ Propafenone ⚬ Amiodarone
- Antibiotics, antiviral, antifungals ⚬ Minocycline ⚬ Isoniazid ⚬ Nitrofurantoin ⚬ Trimethoprim/sulfamethoxazole ⚬ Antiretroviral HIV therapy (combination: emtricitabine, rilpivirine, tenofovir disoproxil fumarate) ⚬ Terbinafine ⚬ Griseofulvin
- Anticonvulsants ⚬ Carbamazepine ⚬ Ethosuximide ⚬ Phenytoin ⚬ Primidone ⚬ Trimethadione
- Antipsychotics and antidepressants ⚬ Chlorpromazine ⚬ Chlorprothixene ⚬ Lithium carbonate ⚬ Phenelzine ⚬ Clozapine ⚬ Bupropion
- Antihypertensives ⚬ Angiotensin-converting enzyme inhibitors (captopril, enalapril, cilazapril) ⚬ Calcium-channel blockers (diltiazem, verapamil, nifedipine) ⚬ Beta-blockers (atenolol, acebutolol, labetalol, pindolol) ⚬ Alpha2-adrenergic agonist (methyldopa, clonidine) ⚬ Alpha1-adrenergic antagonist (prazosin) ⚬ Hydralazine ⚬ Diuretics (hydrochlorothiazide, chlorthalidone) ⚬ Minoxidil
- Antiaggregants (ticlopidine) - Statins (atorvastatin, fluvastatin, lovastatin, pravastatin, simvastatin) - Antithyroid drugs (propylthiouracil) - Proton-pump inhibitors - Immunomodulators ⚬ TNF α inhibitors (infliximab, etanercept, adalimumab) ⚬ Efalizumab ⚬ Interferon α−2a ⚬ Interferon β ⚬ Interleukin-2 ⚬ IgG treatment ⚬ Leflunomide ⚬ Sulfasalazine ⚬ D- Penicillamine
- NSAIDs ⚬ Piroxicam ⚬ Naproxene ⚬ Phenylbutazone
- Checkpoint inhibitors ⚬ Nivolumab ⚬ Ipilimumab
- Chemotherapeutics ⚬ Fluorouracil ⚬ Capecitabine ⚬ Aminoglutethimide ⚬ Mitotane ⚬ Gemcitabine ⚬ Docexatel ⚬ Anastrozole ⚬ Tamoxifen ⚬ Hydroxyurea ⚬ Palbociclib
- Vaccines ⚬ Hepatitis B vaccine ⚬ Influenza vaccine ⚬ Human papilloma virus vaccine ⚬ SARS-CoV2 vaccine
- Miscellaneous ⚬ Levodopa ⚬ Pirfenidone ⚬ Timolol eye drops

DILE is a consequence of breakdown of self-tolerance by disrupting T cell tolerance [28]. Several pathogenic mechanisms have been implicated in the development of DILE, among which the hypomethylation of T cells stands out, leading to increased lymphocytic function that generates autoreactivity, as well as the recently described role of neutrophil extracellular traps or NETosis [7, 29].

The incidence of this condition depends on different factors, such as underlying diseases that require drug associated with the development of lupus. DILE generally affects older patients than idiopathic systemic lupus, and the frequency is similar in both sexes [6]. DILE can be classified into three forms: systemic DILE, drug-induced subacute cutaneous erythematosus (DISCLE) and chronic cutaneous DILE [7]. The onset of symptoms typically occurs months or even years after exposure to causative drug; however, in some cases, it can develop abruptly. The clinical and immunological features of DILE differ from those of idiopathic lupus.

Most common symptoms in systemic DILE include systemic symptoms (low-grade fever, anorexia, weight loss and fatigue), musculo-skeletal symptoms (arthralgia, arthritis or myalgia), skin manifestations [6], pleuritis, pleural effusion, pericarditis, and hepatosplenomegaly [8]. Major organ manifestations are usually absent in DILE, although cases of glomerulonephritis, hematologic abnormalities, central nervous system disease, hepatitis or pneumonitis have been reported [6].

Erythrocyte sedimentation rate (ESR) and C-reactive protein (CRP) are elevated in vast majority of cases of DILE, while cytopenia is not a common finding. Unlike in idiopathic lupus, complement levels are decreased in only one-third of cases. Hypergammaglobulinemia can be present in up to 50% of the cases.

Regarding serological findings, antinuclear antibodies (ANA) are present in 90–100% of cases, typically with specificity for histone proteins, although this is not always the case. DILE with ANA negative and ANA positive without anti-histone antibodies have been reported. Other antibodies have been anecdotally reported, such as anti-dsDNA, ANCA, rheumatoid factor, anticardiolipin, anti-Sm antibodies [8], anti-Ro/SSA and anti-La/SSB [9]. Among the total of sixteen cases of systemic lupus induced by SARS-CoV2 vaccines published up to 2024, the serological pattern is as follows: 100% (16) of the cases tested positive for ANA, 75% (12) for anti-dsDNA, 25% (4) for some antiphospholipid antibody or lupus anticoagulant, only 19% (3) for anti-histone, and 62,5% (10) showed complement pathway involvement [13–26]. Although the number of cases is small to draw definitive conclusions, the serological pattern of vaccine-induced lupus seems to differ from that typically associated with drug-induced lupus: positivity for ANA in all cases, a more frequent anti-dsDNA and antiphospholipid pattern, and a lower frequency of positive anti-histone antibodies.

Unlike systemic lupus erythematosus, DILE typically does not fulfill the diagnostic criteria established by the American College of Rheumatology (ACR). In most cases, patients exhibit only isolated clinical manifestations or meet select serological criteria [6].

Recognizing the offending drug linked to DILE is the first and utmost step in DILE management because DILE resolves after discontinuation of the causative drug, although in some cases, there may be a delay of several months before complete resolution. The treatment of DILE depends on the severity of symptoms: discontinuation of the causative drug could be sufficient, while corticosteroids, antimalarials, anti-inflammatory agents, and immunosuppressants should be reserved for organ damage [9]. Autoantibody titters decline gradually after drug discontinuation and serologic abnormalities can persist positively months after clinical recovery. Upon rechallenge with the offending agent, symptoms recur within 1–2 days [8, 9].

## Conclusion

Drug-induced lupus is challenging because proving causality is difficult, however, the combined temporal association and the clinical manifestation argues for a causative role of Shingrix vaccine in the development of systemic lupus manifestations. Furthermore, special attention should be given to cases of vaccine-induced lupus, as their serological pattern may differ from that typically observed in drug-induced lupus cases.

Our case details a critical adverse effect observed in a patient who received zoster vaccine. This finding is particularly relevant given the ongoing widespread vaccination campaigns and the global public health implications. By publishing this case, we aim to contribute valuable information to the existing literature that can help guide clinicians in identifying, managing, and understanding similar adverse reactions.

This case provides novel insight into the potential role of the Shingrix vaccine in the development of systemic lupus, highlighting a previously underrecognized adverse reaction. The temporal association between vaccination and the onset of lupus manifestations, combined with the distinct serological profile observed, underscores the importance of recognizing vaccine-induced lupus as a differential diagnosis, particularly in the context of widespread vaccination campaigns. Given the global public health impact of zoster vaccination, this finding has significant implications for clinicians, emphasizing the need for vigilance and prompt identification of such adverse reactions. This report adds critical evidence to the growing body of literature, aiding in the refinement of diagnostic and management strategies for vaccine-related lupus, ultimately contributing to safer vaccination practices.

## Data Availability

The data supporting the findings of this case report are available from the corresponding author upon reasonable request. Due to patient privacy, the data are not publicly available.
